# Intestinal Tuberculosis Masquerading as Crohn's Disease? A Case of Disseminated Tuberculosis after Anti-TNF Therapy for Suspected Crohn's Disease

**DOI:** 10.1155/2019/6053503

**Published:** 2019-12-14

**Authors:** Alexander P. Abadir, James Y. Han, Fady A. Youssef

**Affiliations:** University of California Irvine Medical Center, Orange, USA

## Abstract

Intestinal tuberculosis (ITB) and Crohn's disease (CD) very closely resemble each other in symptomatology, imaging, appearance, and pathology. While ITB is rare in the United States, its prevalence is significantly higher in endemic areas, thus presenting a diagnostic dilemma in immigrant populations from high-risk countries. This patient was diagnosed with CD and treated with anti-TNF agents after indeterminate screening for latent tuberculosis. He was then admitted with septic shock and intestinal perforation due to disseminated tuberculosis. This case demonstrates the importance the consideration of ITB when a patient with risk factors for TB fails to respond to treatment for CD.

## 1. Introduction

Crohn's Disease (CD) is classically characterized by inflammation and ulceration at the ileocecal junction. Symptoms of CD include moderate to severe abdominal pain, bloody stool, and weight loss. Intestinal tuberculosis (ITB) can similarly present as inflammation and ulceration at the ileocecal junction. Therefore, ITB can often mimic CD in clinical features and gross pathology.

## 2. Case Report

**Figure 1:**
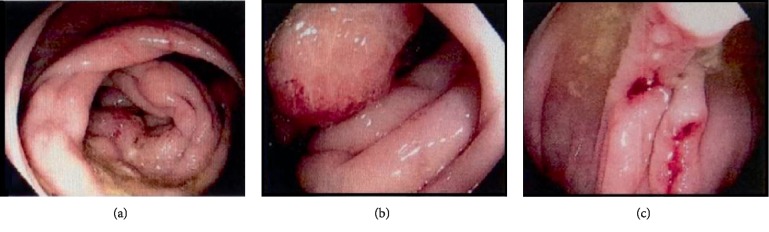
Significant findings from initial colonoscopy which the diagnosis of Crohn's disease was made. (a) Inflammation seen in cecum. (b) Inflammation in ascending colon. (c) Inflammation with friability and hemorrhage at the hepatic flexure.

**Figure 2:**
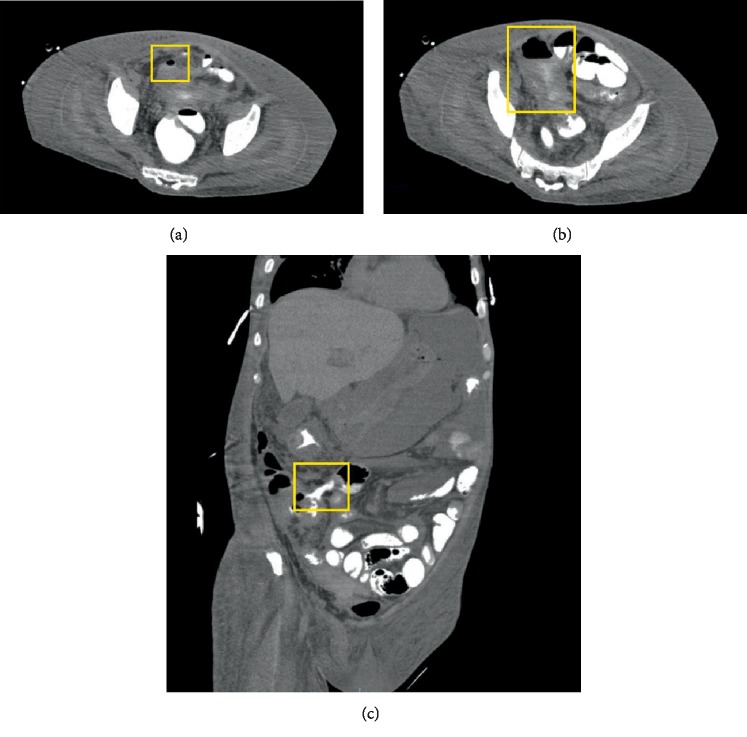
CT scan images of significant findings on hospitalization including (a) Free air in the pelvis. (b) Free air with leaked contrast in the peritoneal space. (c) An enterocolic fistula. These findings determined the decision for exploratory laparotomy.

A 47-year-old male born in the Philippines who immigrated to the US approximately 2 years ago presents to a gastroenterologist with symptoms of abdominal pain, unintentional 60 lb weight loss over the past 6 months, fatigue, and diarrhea without hematochezia. A CT scan of the abdomen showed only nonspecific colitis of the ascending colon and descending colon. He had a colonoscopy which showed segmental areas of inflammation and deep ulcerations at the hepatic flexure and in the ascending colon with edema, granularity, and loss of vascularity (Figure 1). The terminal ileum was not intubated during this procedure. Pathology showed severe lymphoplasmacytic infiltration, marked architectural distortion, and chronic inflammation without granulomas. The patient was diagnosed with Crohn's disease and was treated with prednisone and mesalamine. However, his symptoms progressively worsened over the next three months, so his treatment was escalated. He was tested for latent tuberculosis infection (LTBI) with the Quantiferon gold assay, which returned indeterminate and a subsequent tuberculin skin test (TST) was negative. Chest X-ray did not show any evidence of active or prior TB infection, so the patient was thus presumed to be TB negative and started on the TNF-alpha inhibitor infliximab. One month later, the patient presented with worsening abdominal pain, diarrhea, fatigue, and new fevers, claiming that his symptoms had significantly worsened since starting the infliximab. He was in shock with a blood pressure of 73/51 mmHg and laboratory results were notable for white blood cell count of 8.7 × 10^9^/L (normal 3.4–9.6 × 10^9^/L) with bandemia, albumin 1.5 g/dL (normal 3.5–5.0 g/dL), lactic acid 5.3 mmol/L (normal 0.5–1 mmol/L), and a cholestatic liver function pattern. He was treated for septic shock with broad-spectrum IV antibiotics and vasopressors, but required intubation due to clinical deterioration. CT imaging revealed ascites, large bilateral pleural effusions, and multiple hypodense lesions within the liver with an obstructing mass at the confluence of the bile ducts. Diffuse full-thickness small and large bowel wall thickening along with increased attenuation involving the terminal ileum and cecum was present. A workup for infection was pursued with liver lesion biopsy, thoracentesis, and paracentesis. These studies were negative for malignant cells, but were positive for numerous acid-fast bacilli (AFB). A repeat CT was performed where it was seen that the patient developed intraperitoneal free air and fistulas requiring exploratory laparotomy (Figure 2). Surgery revealed extensive adhesions associated with dense granulomatous disease and perforation of the small bowel and right colon. A hemicolectomy and small bowel excision was performed with pathology of the terminal ileum and right colon showed extensive caseating and noncaseating granulomatous inflammation that stained positive for AFBs in a skip lesion pattern consistent with intestinal tuberculosis (ITB). The patient was diagnosed with disseminated TB and started on anti-mycobacterial therapy with significant improvement in his clinical condition. After discharge, he no longer complained of diarrhea, weight loss, or abdominal pain on two month follow-up. The diagnosis of Crohn's Disease was no longer considered and removed from his medical history.

## 3. Discussion

ITB can mimic inflammatory intestinal diseases such as Crohn's Disease, and in the absence of other clinical manifestations of TB, differentiating between primary ITB and CD can be a significant diagnostic challenge. The diagnosis of ITB is rare in developed countries such as the United States and accounts for less than 1% of all cases of abdominal tuberculosis [1]. However, its prevalence is significantly higher in countries where tuberculosis is endemic, such as India, African, and Southeast Asia. Differentiating between these two diagnoses is critical because the treatments are radically different, and administering immunosuppressive medications to a patient with ITB misdiagnosed as CD can be fatal.

Both diseases can present with clinical symptoms of weight loss, abdominal pain, fever, bowel obstruction, and bloody diarrhea, and endoscopic findings of skip lesions, ulcerations, and terminal ileum involvement. The histologic hallmark of ITB that best distinguishes it from CD is confluent caseating granulomas within the submucosa with positive AFB staining, though this is seldom seen and there is enough variability between pathologists' interpretations such that there have been reports of patients being diagnosed with Crohn's Disease only to have their diagnosis changed to ITB upon review of pathology specimens by a second pathologist [2–5].

The diagnosis of ITB requires a high degree of suspicion, especially when encountering a patient who immigrated from an endemic area, and the clinician must consider the patient's background, prior history of gastrointestinal and pulmonary infections, family history of GI diseases, and personal contacts with people who may have had TB. TB testing with QuantiFERON or TST should be considered in all patients who immigrated from TB endemic areas. For this patient, antitubercular therapy would have been a reasonable first choice for therapy given his background of recent immigration from the Philippines. For patients who are started on steroids, a lack of response should prompt consideration of an alternative diagnosis, which includes a variety of infections such as ITB and CMV colitis. Appropriate strategies for reevaluation can include repeating a CT scan, testing for TB, repeating a colonoscopy with terminal ileum intubation, or a trial of antituberculosis therapy. Although most patients who test negative with a TST and clear CXR can safely proceed to TNF-a inhibitors, patients who have TST performed while on steroids and in whom clinical suspicion is high for TB may benefit from repeat testing performed with a QuantiFERON test.

Unfortunately ITB remains an underrecognized disease in nonendemic countries such as the United States and can lead to life-threatening misdiagnosis such as in this case. Clinicians should always keep in mind which areas are endemic for TB when evaluating immigrant patients with gastrointestinal symptoms suspicious for CD and consider early TB testing. Furthermore, clinicians must always consider the alternative diagnosis of ITB if standard treatment for CD does not yield the appropriate response and consider repeating imaging and endoscopic procedures to confirm the original diagnosis before committing the patient to powerful immunosuppressant medications.
